# Antimicrobial Resistance in *Salmonella enterica* Serovar Paratyphi B Variant Java in Poultry from Europe and Latin America

**DOI:** 10.3201/eid2606.191121

**Published:** 2020-06

**Authors:** L. Ricardo Castellanos, Linda van der Graaf-van Bloois, Pilar Donado-Godoy, Kees Veldman, Francisco Duarte, María T. Acuña, Claudia Jarquín, François-Xavier Weill, Dik J. Mevius, Jaap A. Wagenaar, Joost Hordijk, Aldert L. Zomer

**Affiliations:** Utrecht University, Utrecht, the Netherlands (L.R. Castellanos, L. van der Graaf-van Bloois, D.J. Mevius, J.A. Wagenaar, J. Hordijk, A.L. Zomer);; Corporación Colombiana de Investigación Agropecuaria–AGROSAVIA, Cundinamarca, Colombia (P. Donado-Godoy);; Wageningen Bioveterinary Research, Lelystad, the Netherlands (K. Veldman, D.J. Mevius, J.A. Wagenaar);; Instituto Costarricense de Investigación y Enseñanza en Nutrición y Salud, Tres Ríos, Costa Rica (F. Duarte, M.T. Acuña);; Universidad del Valle de Guatemala, Guatemala City, Guatemala (C. Jarquín);; Institut Pasteur, Paris, France (F.-X. Weill)

**Keywords:** Broiler, whole-genome sequencing, molecular clock, Bayesian skyline, pAmpC, CMY-2, qnrB19, IncI1, IncHI2, Salmonella enterica serovar Paratyphi B variant Java, Europe, Latin America, antimicrobial resistance, bacteria

## Abstract

*Salmonella enterica* serovar Paratyphi B variant Java sequence type 28 is prevalent in poultry and poultry meat. We investigated the evolutionary relatedness between sequence type 28 strains from Europe and Latin America using time-resolved phylogeny and principal component analysis. We sequenced isolates from Colombia, Guatemala, Costa Rica, and the Netherlands and complemented them with publicly available genomes from Europe, Africa, and the Middle East. Phylogenetic time trees and effective population sizes (*N_e_*) showed separate clustering of strains from Latin America and Europe. The separation is estimated to have occurred during the 1980s. *N_e_* of strains increased sharply in Europe around 1995 and in Latin America around 2005. Principal component analysis on noncore genes showed a clear distinction between strains from Europe and Latin America, whereas the plasmid gene content was similar. Regardless of the evolutionary separation, similar features of resistance to β-lactams and quinolones/fluoroquinolones indicated parallel evolution of antimicrobial resistance in both regions.

The d-Tartrate fermenting, nonparatyphoidal variant of *Salmonella enterica* serovar Paratyphi B, contemporarily known as variant Java, was first reported in 1935 by De Moor (*1*). From then until recently, *Salmonella* Paratyphi B var. Java caused sporadic self-limiting gastrointestinal infections in humans (*2*–*4*). Since 1990, reports of human infections increased in Europe (*5*–*10*), North America (*11*–*15*), and Australia (*16*,*17*). Human infections with this serovar were mainly related to exposure to fish or reptiles (*16*–*18*). A dramatic increase in prevalence in poultry and poultry meat in Europe was reported (*19*,*20*), accompanied by an increase in antimicrobial resistance of the strains (*5*,*19*).

Using a multilocus sequence typing (MLST) scheme for *S. enterica* (*21*), strains of *Salmonella* Paratyphi B var. Java were classified into different sequence types (STs). In a previous study, the use of both traditional serotyping and MLST was instrumental in identifying reptiles as the main source of *Salmonella* Paratyphi B var. Java infections in humans in Germany. Isolates originating from poultry were strongly associated with ST28, those from reptiles with ST88, and those from humans mainly with ST43 and ST149 (*18*). Connor et al. confirmed association of *Salmonella* Paratyphi B var. Java ST28 with samples originating from poultry with whole-genome sequence (WGS) data (*22*). Isolates of *Salmonella* Paratyphi B var. Java from poultry and food products have been identified as carriers of *mcr* gene variants conferring resistance to colistin (*23*,*24*) and class 1 integrons in conjugative plasmids (*5*). Furthermore, near-identical plasmids carrying genes conferring resistance to third-generation cephalosporins were identified in *Escherichia coli*, *Salmonella* Paratyphi B var. Java ST28, and other *Salmonella* serovars known to cause infections in humans, such as Heidelberg and Enteritidis (*25*). In these regards, poultry-associated ST28 is of public health concern because it can be a reservoir of antimicrobial resistance to other *Salmonella* serovars of relevance to humans and species of *Enterobacteriaceae* in poultry and food products.

Previous reports from Latin America countries have identified *Salmonella* Paratyphi B var. Java as highly prevalent in poultry and poultry meat (*26*–*30*). Comparisons between strains of *Salmonella* Paratyphi B var. Java ST28 from Colombia and Europe showed a phylogenetic separation between isolates from Colombia and Europe (*31*). We then hypothesized that the separate lineage of Colombia strains could be part of a larger lineage of *Salmonella* Paratyphi B var. Java ST28 circulating in Latin America (*31*). Further investigation of this hypothesis could help identify potential events in poultry management (e.g., farming and trade) leading to the emergence and successful spread of *Salmonella* Paratyphi B var. Java ST28 in both regions. Our objective was to compare the evolutionary and antimicrobial resistance relatedness of poultry-associated *Salmonella* Paratyphi B var. Java ST28 from Europe and Latin America using WGS-based phylogenetic and temporal analysis.

## Methods

### Strain and Genome Collection

Our study comprised isolates from countries in Latin America and Europe that were previously serotyped as *Salmonella* Paratyphi B var. Java. We selected strains from different countries as follows.

#### Colombia

We used 259 epidemiologically independent isolates from broilers and broiler meat. We obtained isolates from baseline studies in poultry conducted in Colombia during 2008–2013 that originated from 25 samples from farms, 49 samples from slaughterhouses, and 185 samples from retail meat (*32*). As a rule of thumb, 20% of these isolates were randomly sampled using the select cases option in SPSS Statistics 24 (IBM, https://www.ibm.com). As a result, we selected 52 isolates, 5 originating from samples from farms, 5 from samples from slaughterhouses, and 42 from samples from retail.

#### Costa Rica

We selected all available strains from a previous study to determine the prevalence of *Salmonella* spp. in chickens at slaughter in the country in 2009 (*33*). The Costa Rican Institute for Research and Training in Nutrition and Health (Tres Ríos, Costa Rica) made 15 isolates available and provided 3 nonbroiler strains of human, reptile, and swine origin.

#### Guatemala

Available strains from a previous study to determine the prevalence of *Salmonella* in retail raw chicken carcasses in the country were included (*29*). The Center for Health Studies from the Universidad del Valle de Guatemala (Guatemala City, Guatemala) made 5 isolates available.

#### The Netherlands

Isolates were collected in the Netherlands as part of the Monitoring of Antimicrobial Resistance and Antibiotic Usages in Animals program (*34*). Our study comprised 1,279 isolates obtained during 2000–2016. The isolates originated from broilers, broiler meat, and chicken products (1,100 isolates), human enteric infections (159 isolates), and other animals and food items (20 isolates). A stratified random sampling was performed with the Transform, Rank and Select Cases options in SPSS Statistics 24 for every year within the samples from broilers and broiler meat. In addition, we included 2 randomly selected isolates from the entire pool of human isolates and 2 from other animals and food items as nonbroiler references. As a result, 21 isolates originating from 16 broilers and 1 broiler meat sample, 2 human enteric infections, and 2 from other animals and food products were selected.

#### Historical *Salmonella* Paratyphi B Var. Java ST28 Strains

Two strains of human origin from Saudi Arabia isolated in 1987 (IP_6155/87) and 1992 (IP_7734/92) and 1 strain from a turkey of Israel origin isolated in Austria in 1988 (IP_6395/88) were identified from their MLST profiles. These strains were the earliest *Salmonella* Paratyphi B var. Java ST28 strains from the EnteroBase database (https://enterobase.warwick.ac.uk) (*35*).

#### Publicly Available WGS Sequences

We queried EnteroBase for assembled genomes using the “experimental data” search option for strains belonging to “ST28” in the “Achtman 7-gene MLST” scheme (accessed February 22, 2018). We selected 65 strains with metadata available for year and country of isolation for phylogenetic and temporal analysis (Appendix 1).

### WGS and In Silico Screening of Antimicrobial Resistance Genes and Plasmid Content

We isolated genomic DNA from selected strains from the Netherlands, Colombia, Costa Rica, and Guatemala using the UltraClean Microbial DNA Isolation Kit (QIAGEN, https://www.qiagen.com). We performed WGS on the MiSeq platform using 2 × 250-bp reads and the NextSeq platform using two 2 × 150-bp reads (Illumina, https://www.illumina.com). WGS of historical strains was performed at the Plateforme de microbiologie mutualisée from the Pasteur International Bioresources network (PIBnet, Institut Pasteur, https://www.pasteur.fr) as previously described (*36*). We assembled the genomes of historical and newly sequenced strains with SPAdes version 3.10.1 (*37*) and screened for antimicrobial resistance genes and chromosomal mutations using ResFinder 3.1 (*38*). We subtyped plasmids using PlasmidFinder 2.0 and plasmid MLST (pMLST) 2.0 (*39*). For newly sequenced genomes, we performed 7-gene MLST at the strain level with MLST 1.8 (*40*).

### Phylogenetic Time Trees and Effective Population Size Estimates

For phylogenetic single-nucleotide polymorphism (SNP) analysis of the core genome, we aligned WGS of all *Salmonella* Paratyphi B. var. Java ST28 isolates using Parsnp version 1.2 (*41*), excluding non-ST28 strains. We used Gubbins (*42*) to detect and visualize recombination regions in the core genome alignment and performed time-resolved phylogeny on recombination-filtered SNPs of the *Salmonella* Paratyphi B. var. Java ST28 isolates that were extracted from the Gubbins results and used for divergence dating in BEAST (*43*). We included only newly obtained genomes with coverage >30. We used isolation dates as tip dates in the phylogenetic tree, as outlined in the BEAST manual, with the following modifications: 10,000,000× sampling and a general time-reversible model plus γ correction as the distance model. We tested a strict clock, relaxed logarithmic clock, and relaxed exponential clock as the clock model. We used a Bayesian skyline plot with 3 groups as demographic models to adjust for expected population changes and the effective sample size (ESS) method to select the strict clock model because it had the highest ESS values. All ESS values obtained with the strict clock model were >1,000. To generate Bayesian skyline plots for the European and Latin American populations, we repeated time-resolved phylogeny analysis using BEAST on 2 subsets containing the historical isolates and the isolates from Europe and Latin America.

### Orthology Prediction and Plasmid/Chromosome Contig Scoring

We annotated genomes using Prokka version 1.13 (*44*), followed by orthology predictions using Roary (*45*). We differentiated chromosome and plasmid contigs with an in-house built tool. In brief, we scored contigs for the presence of known plasmid genes (https://github.com/aldertzomer/RFPlasmid), single-copy chromosomal marker genes (*46*), and kmer profiles and inferred their likely origin (plasmid or chromosomal) using a Random Forest model trained on known plasmid and chromosome assemblies (A.L. Zomer, unpub. data).

### Comparison of Accessory Genome

We conducted principal component analysis (PCA) for all isolates on the gene presence/absence tables from the output of orthology predictions. We made comparisons using the accessory (noncore) genome of chromosome contigs, complete plasmid composition with all plasmid contigs, and plasmid composition with only plasmid contigs >50 kb. We made additional characterizations of prophage sequences using the PHAge Search Tool Enhanced Release (*47*) and BLAST (https://blast.ncbi.nlm.nih.gov/Blast.cgi).

### Data Availability

We deposited sequences of the historical and newly sequenced strains in the short-read archive of the European Nucleotide Archive (ENA) under project no. PRJEB31547. Accession numbers of all collected genomes are provided (Appendix 1 Table 1).

## Results

### Phylogenetic Time Tree and Effective Population Size of *Salmonella* Paratyphi B var. Java ST28

Most of the strains from poultry that we sequenced from Colombia (48/52), Costa Rica (15/15), Guatemala (4/5), and the Netherlands (17/17) belonged to ST28 (Table). Similarly, nonbroiler strains from a fish product and a turkey in the Netherlands and 1 from a pig carcass in Costa Rica belonged to ST28. Isolates from 3 human and 1 reptile samples from Costa Rica or the Netherlands belonged to STs different from ST28 (Appendix 1 Table 1).

**Table:** Newly obtained and publicly available genomes of 155 *Salmonella enterica* serovar Paratyphi B variant Java sequence type 28

Source	**Total**	No. per source*	**Years isolated**
Historical			
Saudi Arabia†	2	2 human	1987–1992
Austria†	1	1 poultry‡	1988
Europe			
Belgium§	5	5 unknown	2014
Denmark§	9	8 poultry, 1 unknown	2009–2015
Germany§	5	3 poultry, 1 human, 1 unknown	2001–2013
Ireland§	2	2 human	2015–2016
Nigeria§¶	1	1 poultry	2009
The Netherlands†	19	18 poultry, 1 fish	2000–2016
United Kingdom§	24	18 unknown, 5 human, 1 bovine	2006–2017
Latin America			
Colombia†#	67	67 poultry	2008–2013
Costa Rica†	16	15 poultry, 1 swine	2009–2014
Guatemala†	4	4 poultry	2012

*Source-metadata of publicly available genomes was obtained from EnteroBase (https://enterobase.warwick.ac.uk). †Newly obtained. ‡Sample from a turkey imported from Israel. §Publicly available. ¶Phylogenetically related to the European clade. #19 genomes from previous reports in Colombia were publicly available in EnteroBase (*31*).

**Figure 1:**
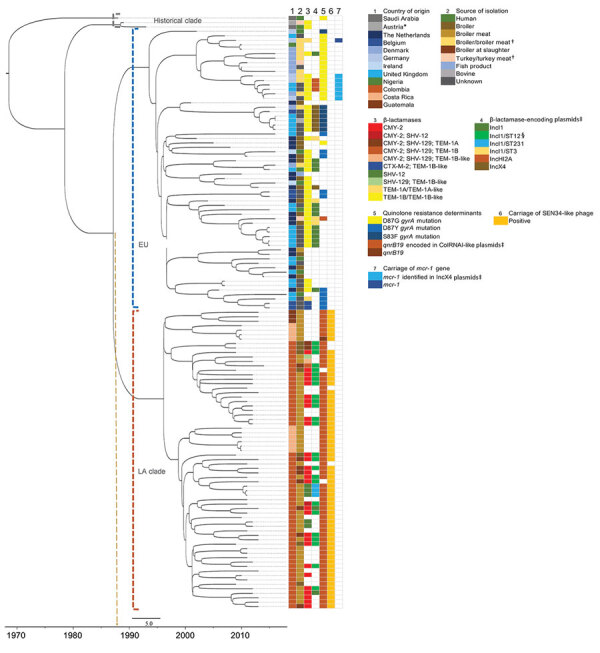
Phylogenetic time tree showing the separation between the historical, EU, and LA clades. Gray brackets indicate historical clades; blue brackets, EU clades; orange brackets, LA clades. Tips in the tree are aligned to the year of isolation of the strains. Nodes are dated in the *x*-axis as estimated by BEAST (*43*). Arrow indicates the node and year of separation between EU and LA clades around 1987. *Sample from a turkey imported from Israel. †Undefined sample material. ‡Plasmids are indicated when resistance genes and plasmid replicons were found in the same contig. §IncI1/ST12 plasmids encoded CMY-2 only. EU, European; LA, Latin American. Scale bar indicates number of years.

**Figure 2:**
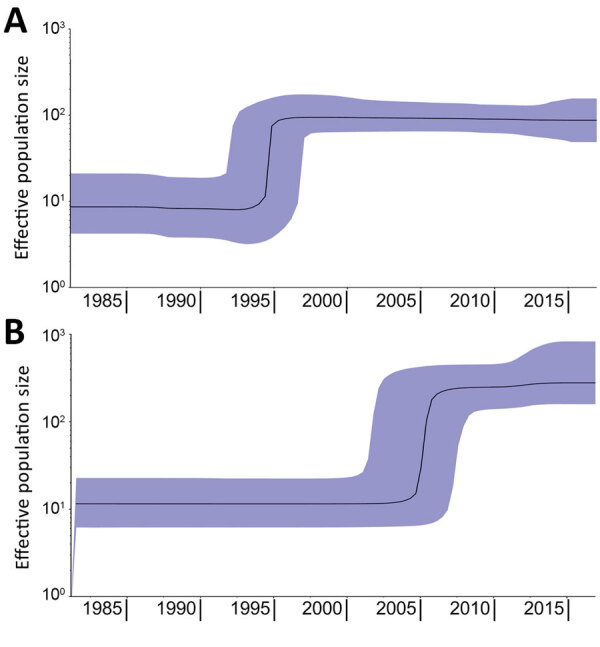
Bayesian skyline plots showing increase in effective population size of *Salmonella enterica* serovar Paratyphi B variant Java sequence type 28. Plots were made separately with strains originating from Europe (A) or Latin America (B). Emergence in Europe occurred in ≈1995 and in Latin America in ≈2005. Black lines indicate estimates of the median population over time; purple shading indicates 95% CIs.

In the phylogenetic trees, the strains collected from the Latin America countries formed a separate cluster. In contrast, the strains collected from the Netherlands clustered with strains originating from other countries in Europe (Figure 1). An additional cluster was formed by the historical strains, which were neither from Europe nor Latin America. The molecular clock was estimated at 3.5 × 10^–7^ substitutions/site/year (1.7 SNPs/genome/year, 95% CI 1.44–2.0 SNPs/genome/year). The output of BEAST indicated separation between strains from Europe and Latin America occurred in 1987 (95% CI 1978–1988) (Figure 1; Appendix 2 Figure 1). From the Bayesian Skyline plot, we inferred that the effective population size (*N_e_*) of strains from Europe increased sharply in 1995 (95% CI 1992–1998) and in Latin America in 2005 (95% CI 2001–2007) (Figure 2), 10 years later than in Europe.

### In Silico Characterization of Frequent Antimicrobial Resistance Genes and Plasmid Subtypes

We found a chromosomal class 2 integron (with *dfrA1*-*sat1*-*aadA1* [GenBank accession no. AB188271.1]) in historical strain IP_6155/87, collected in 1987 in Saudi Arabia. However, this integron was not in the other 2 historical strains from Austria (originating from Israel [IP_6395/88]) and Saudi Arabia [IP_7734/92]). In strain IP_6155/87, the alignment of the class 2 integron was divided into multiple contigs. We also found the integron in all strains from Europe and Latin America in contigs of ≈50 kb carrying the complete integron or divided into multiple contigs. Alignments of ≈50 kb contigs carrying the complete integron, revealed 100% identity within and between strains from European and Latin American clades. 

Resistance to β-lactams in strains from Europe was mainly mediated by *bla*_TEM-1B_ or *bla*_TEM-1B_-like genes. These genes were found in 64% of isolates in the European clade. In strains from the Latin American clade, *bla*_CMY-2_ was most prevalent and was carried in 50% of strains (Figure 1). *bla*_TEM-1B_ gene is known to confer resistance to aminopenicillins and *bla*_CMY-2_ gene to extended-spectrum cephalosporins. In the European clade, *bla*_TEM-1B/1B_-like was mainly found on IncI1 plasmids (17/42) and, to a lesser extent, on IncX4 (7/42) and IncHI2 (3/42) plasmids. In strains from the Latin American clade, *bla*_CMY-2_ was mainly found on IncI1/ST12 plasmids (25/33) (Figure 1). The *sul2* gene, conferring resistance to sulphonamides, was frequently encountered in strains from Europe (48%) and Latin America (14%). In the European clade, *sul2* was mainly found to co-localize with *bla*_TEM-1B/1B_-like on IncI1 plasmids (17/30), whereas in the Latin American clade, *sul2* was found on ColRNAI-like plasmids (9/9). β-lactam and sulphonamide resistance genes in the Latin American clade were observed only in strains from Colombia (Figure 1). In addition, >50% of strains from the European clade exhibited known DNA gyrase mutations conferring resistance to fluoroquinolones. The observed mutations were *gyrA* D87G (16/33), D87Y (10/33), and S83F (7/33) (Figure 1). Although no strains from the Latin American clade carried the chromosomal *gyrA* mutations, they did carry *qnrB19*-harboring plasmids in 96% of the cases. *qnrB19* is known to confer reduced susceptibility to quinolones. This gene was co-localized with ColRNAI-like plasmids in strains from Latin America and in 1 strain from Europe from recent years (2015) (Figure 1).

### PCA of Accessory Genomes

**Figure 3:**
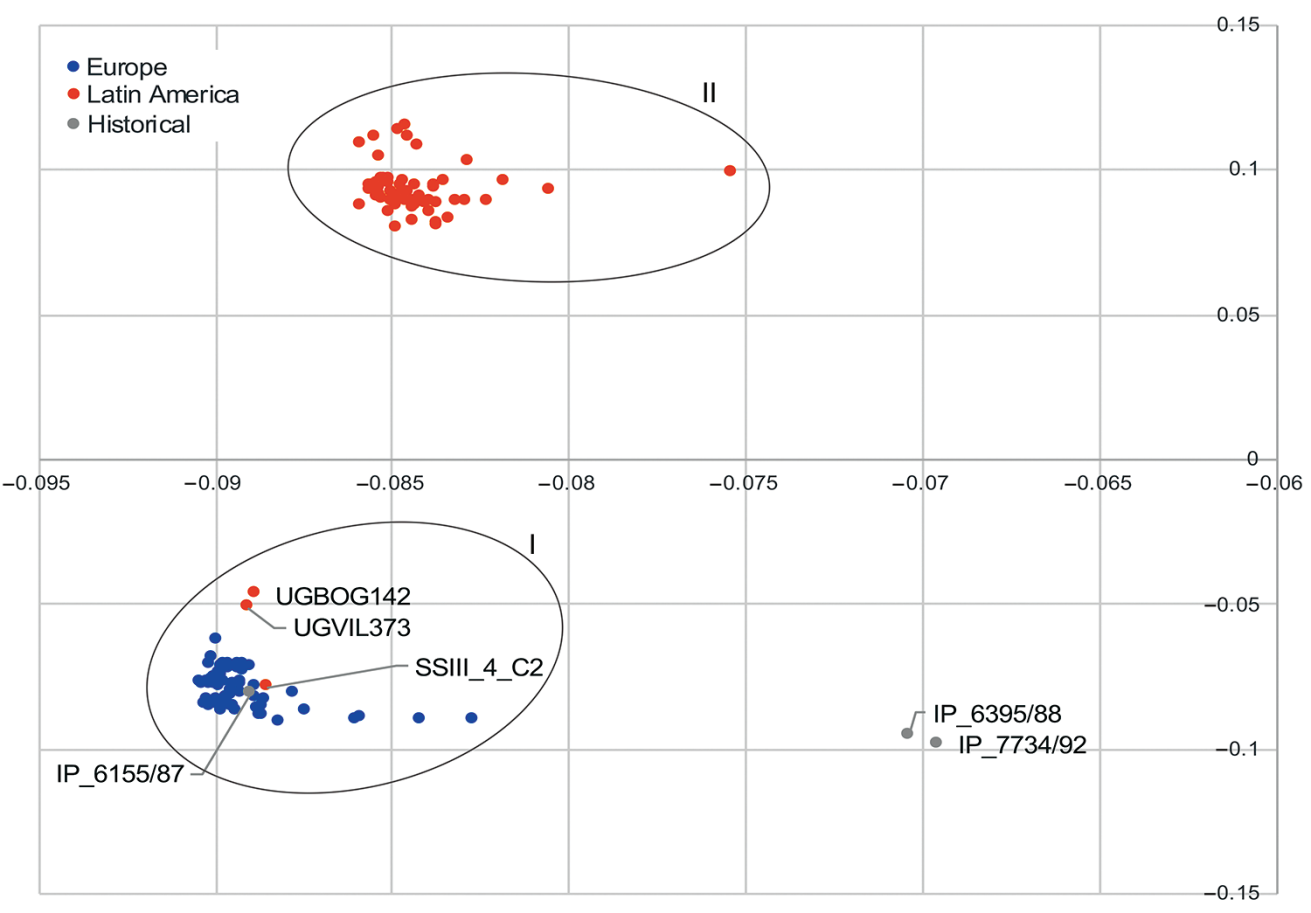
Principal component analysis plot comparing accessory (noncore) genome of chromosome contigs of strains of *Salmonella enterica* serovar Paratyphi B variant Java sequence type 28. Oval rings indicate clusters I and II. Cluster I grouped together historical strain IP_6155/87 with all strains from Europe and some from Latin American. Cluster II grouped Latin America strains only. Cluster II was associated with a prophage sequence highly similar to the *Salmonella* phage SEN34 (National Center for Biotechnology Information reference sequence no. NC_028699.1). The prophage sequence was absent in strains from cluster I.

**Figure 4:**
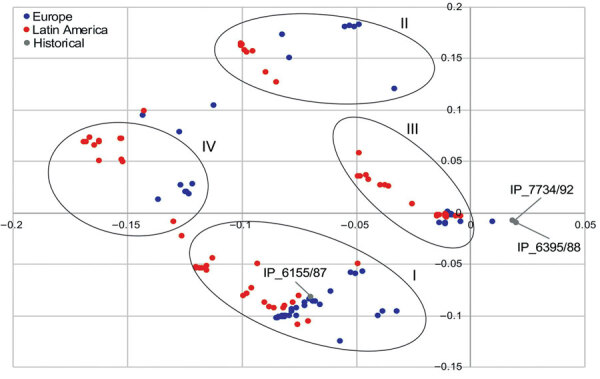
Principal component analysis plot comparing plasmid composition (all plasmid contigs) of strains of *Salmonella enterica* serovar Paratyphi B variant Java sequence type 28. Oval rings indicate clusters I–IV. All clusters grouped strains from Europe and Latin America and were associated with IncI1 plasmids (cluster I), IncHI2 (cluster II), COLRNAI (cluster III), and combinations of IncI1 and IncHI2 plasmids (cluster IV).

We observed a marked distinction for the accessory genes located on chromosome contigs between the European and Latin American clades (Figure 3). The separation in the PCA was associated with the presence of a prophage highly similar to the *Salmonella* phage SEN34 (National Center for Biotechnology Information reference sequence NC_028699.1) in strains from Latin America. This phage was also found in the genome of a *Salmonella* serovar Saintpaul strain submitted in Canada (GenBank accession no. CP022491.1). A few strains from Colombia closer to the cluster of strains from Europe in the PCA (UGBOG142, UGVIL373, and SSIII_4_C2) lacked the sequence of this phage. Plasmid composition was similar in strains from Europe and Latin America (Figure 4). The profiles of plasmid composition were characterized by the presence of IncI1 plasmids (cluster I), IncHI2 (cluster II), ColRNAI-like (cluster III), and combinations of IncI1 and IncHI2 plasmids (cluster IV). Although the IncI1 plasmids had different pMLST sequence types, their content appears to be remarkably similar as they are in proximity in the PCA plot (cluster I). When exploring this further using only plasmid contigs >50 kb in strains from both European and Latin American clades, we observed that the IncI1 plasmid contigs clearly have near identical content (cluster I in Appendix 2 Figure 2). In strains with multiple plasmid contigs, in addition to the cluster of IncI1-like plasmids, IncHI2-like plasmid contigs were differentiated in cluster II (Appendix 2 Figure 2).

## Discussion

We selected public genomes using EnteroBase and based on metadata availability and in silico serovar and MLST characterization. In a comparison between EnteroBase, ENA, and GenBank (Appendix 1 Table 2), we found 3,524 genomes associated to BioSamples (i.e., unique identifiers linking metadata and sequencing data) after querying EnteroBase for predicted serovar “Paratyphi B Var. Java.” After querying “*Salmonella* AND Java,” we found 1,245 in ENA and 1,299 in GenBank. This difference is mainly due to the large number of genomes submitted without serovar information (1,935/3,524) rather than those labeled differently from Paratyphi B or variant Java (71 genomes). In this regard, in silico characterization in EnteroBase helped surpass absent, incorrect, or multiple serovar denominations accompanying genomes submitted to the ENA and GenBank databases.

On the basis of MLST, we found that poultry-associated variant Java ST28 (380 isolates), human-associated ST43 (767 isolates), and reptile-associated ST88 (319 isolates) were among the most abundant STs in EnteroBase. Most (143 isolates) ST28 genomes originated from poultry, and some (24 isolates) originated from humans, which could indicate transfer between these 2 sources. Because metadata were lacking on their year of isolation in EnteroBase (Appendix 1 Table 2), some ST28 genomes were excluded from the phylogenetic time trees in this study. Nevertheless, phylogenetic analysis based on core genome MLST in EnteroBase (*35*) strongly supported the separate clustering of historical strains and those from Europe and Latin America (Appendix 2 Figure 3).

From the time-resolved phylogeny, we observed that strain IP_6155/87 collected in 1987 was ancestral to all the other strains analyzed. IP_6155/87 can be hypothesized to be the strain most closely related to the common ancestor of all analyzed strains, which according to the output from the time-resolved phylogeny circulated around 1970 (95% CI 1962–1974). This hypothesis is supported by the high level of similarity between the contigs carrying the class 2 integron (with *dfrA1*-*sat1*-*aadA1* [GenBank accession no. AB188271.1]) between historical strain IP_6155/87 and strains from Europe and Latin America. Common ancestry of IP_6155/87 was also reflected in the PCAs used to compare the accessory genome. At both chromosome and plasmid levels, the accessory genome of this strain was closely related to strains from Europe, Latin America, or both (Figures 3, 4; Appendix 2 Figure 2).

In comparison with previous estimations of mutation rates for *Salmonella* Typhimurium DT104 (3.4 × 10^−7^ substitutions/site/year) (*48*), the molecular clock calibrated for *Salmonella* Paratyphi B var. Java ST28 in our study was estimated at 3.5 × 10^–7^ substitutions/site/year, corresponding to 1.7 SNP/genome/year (95% CI 1.44–2.0 SNP/genome/year). Our results indicate similar mutation rates for these 2 *S. enterica* serovars with distinct ecologic niches.

Previously, genomic characterization of *Salmonella* Paratyphi B var. Java ST28 from Colombia suggested a different clade from the one observed in Europe could be circulating in Latin America (*31*). We found a distinct clade of *Salmonella* Paratyphi B var. Java ST28 circulating in poultry from Costa Rica, Guatemala, and Colombia. In Colombia, introduction of foreign technologies for poultry breeding, housing, and processing occurred around 1960 (*49*). Importation of this particular *S. enterica* serovar was anticipated to have occurred around this time. Nevertheless, separation between the European and Latin American clades in our study was estimated with BEAST in the 1980s (Figure 1; Appendix 2 Figure 1). Furthermore, an increase in effective population size in Latin America was observed only in 2005 (95% CI 2001–2007), 10 years after the known increase in Europe, reported in the literature (*19*) and observed with Bayesian skyline in our study (Figure 2). Driving factors that led to the separation of clusters could not be determined based on the availability of data for this study.

The separation between the Latin American and European clades comprised differences at both the core and noncore genome level. Differences in antimicrobial resistance gene content, plasmid replicons, and pMLSTs reflected the evolutionary separation of the 2 clades. Among these differences, DNA gyrase mutations conferring resistance to fluoroquinolones and *bla*_TEM-1B_–carrying IncI1 plasmids were characteristic in the European clade. In contrast, *qnrB19*-carrying ColRNAI-like plasmids conferring reduced susceptibility to quinolones and *bla*_CMY-2_–carrying IncI1/ST12 plasmids were found in Latin America. In both clades, resistance to β-lactams was mainly carried on IncI1 plasmids, with near-identical gene content (cluster I in Figure 4) but from different pMLST lineages. *bla*_TEM-1B_ in Europe was associated with IncI/ST3 in some strains and *bla*_CMY-2_ with IncI1/ST12 in Latin America. It is remarkable that the genomic features confer resistance to β-lactam drugs and quinolones/fluoroquinolones in both European and Latin American clades and thus indicate parallel evolution of *Salmonella* Paratyphi B var. Java ST28 in both geographic regions. Acquisition of such antimicrobial resistance traits can be hypothesized to have occurred as a consequence of selection pressure posed by the use of β-lactams and fluoroquinolones in poultry production. As an example, and as previously suggested, the use of aminopenicillins in broiler production potentially could have selected for *bla*_CMY-2_–carrying *E. coli* strains in the absence of the use of cephalosporins (*50*). Furthermore, the emergence of *Salmonella* Paratyphi B var. Java in Europe was associated with increased antimicrobial resistance and the use of fluoroquinolones, such as flumequine (*19*) or enrofloxacin, which could also explain the sharp increase in effective population size around 1995. For Latin America, such information is not available because data on the use of antimicrobial drugs in animals is not systematically collected in most countries in the region.

In conclusion, *Salmonella* Paratyphi B var. Java ST28 from poultry in Europe and Latin America form 2 different clades. The separation is estimated to have occurred during the 1980s (95% CI 1978–1988). The years with sharp increase in effective population size were estimated as 1995 for Europe and 2005 for Latin America. Previous reports about the emergence of *Salmonella* Paratyphi B var. Java in poultry in Europe supports these findings for Europe (*6*,*19*); no historical data are available for Latin America. In spite of their evolutionary divergence, the European and Latin American clades have independently acquired different antimicrobial resistance genes on similar plasmids. These genetic determinants confer resistance to β-lactam drugs and quinolones and thus indicate parallel evolution of *Salmonella* Paratyphi B var. Java ST28 in both regions.

Appendix 1 Additional information in a study of antimicrobial resistance in *Salmonella enterica* serovar Paratyphi B variant Java.

Appendix 2 Polygenic time tree and principle component analysis plots of a study of antimicrobial resistance in *Salmonella enterica* serovar Paratyphi B variant Java.
