# Supplementary material for: Antimicrobial Resistance in Salmonella enterica Serovar Paratyphi B Variant Java in Poultry from Europe and Latin America
Source: Emerg Infect Dis. 2020 Jun;26(6):1164–73. doi: 10.3201/eid2606.191121 (PMC7258445; doi:10.3201/eid2606.191121)
Supplement: Appendix 2 — Polygenic time tree and principle component analysis plots of a study of antimicrobial resistance in Salmonella enterica serovar Paratyphi B variant Java. [file 19-1121-Techapp-s2.pdf]

Article DOI: https://doi.org/10.3201/eid2606.191121

Antimicrobial Resistance in *Salmonella enterica* Serovar Paratyphi B Variant Java in Poultry from Europe and Latin America

Appendix 2

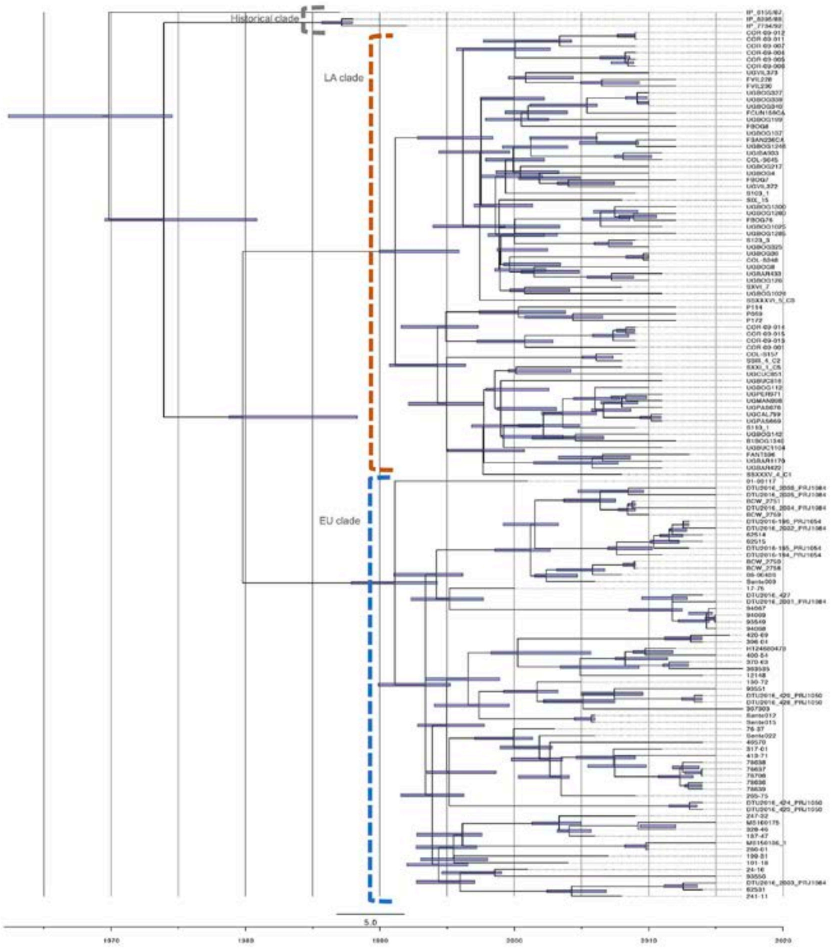

Appendix 2 Figure 1. Phylogenetic time tree with confidence intervals showing the phylogenetic separation between the historical, LA, and EU clades. Gray brackets indicate strains from historical clades; orange brackets, strains from LA clades; and blue brackets, strains from EU clades. Scale bar represents number of years. Tips in the tree are aligned to the year of isolation of the strains. Nodes are dated in the x-axis as estimated by BEAST (*1*). Horizontal blue bars at the nodes represents 95% CI for each estimate. EU, European; LA, Latin American.

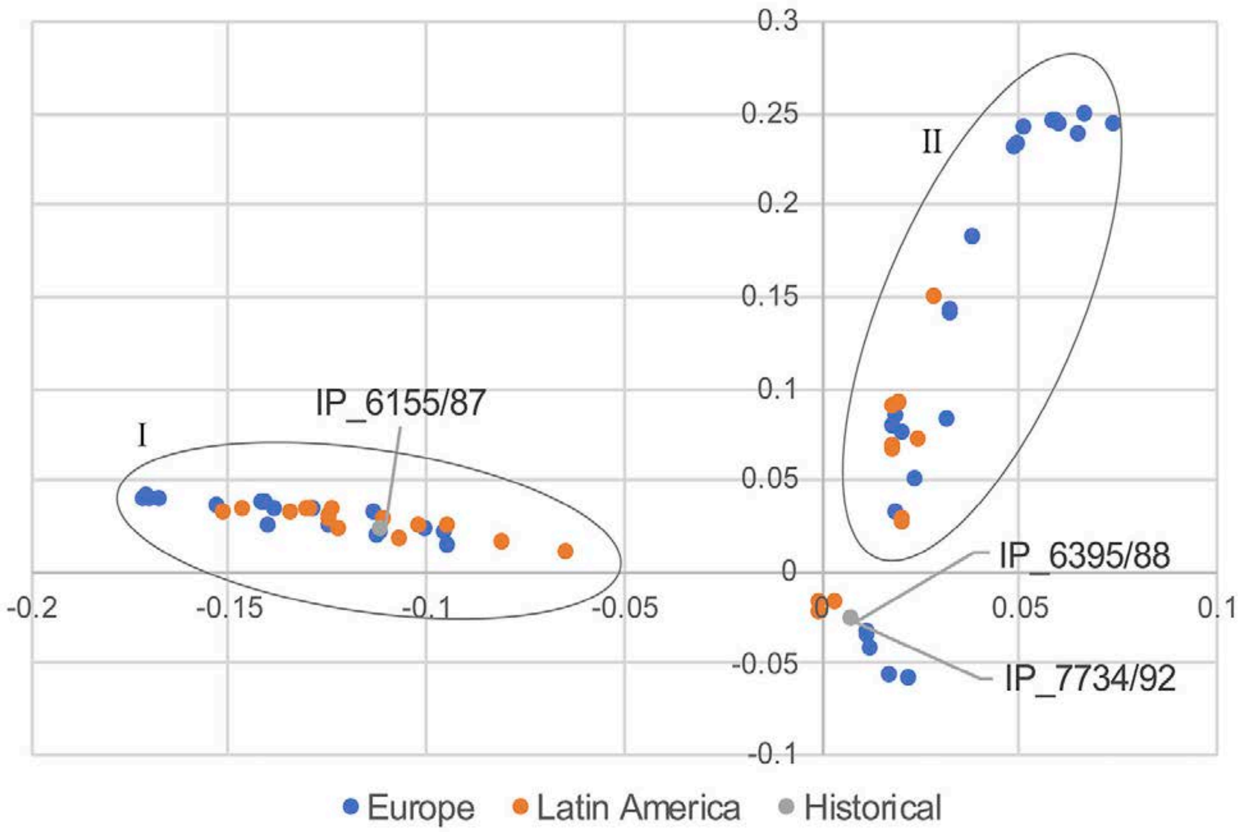

Appendix 2 Figure 2. Principal component analysis plot comparing plasmid composition (plasmid contigs >50 kb only) of strains of *Salmonella enterica* serovar Paratyphi B variant Java sequence type 28. Clusters I and II are indicated by oval rings and grouped IncI1 and IncHI2 plasmids with near identical content, respectively.

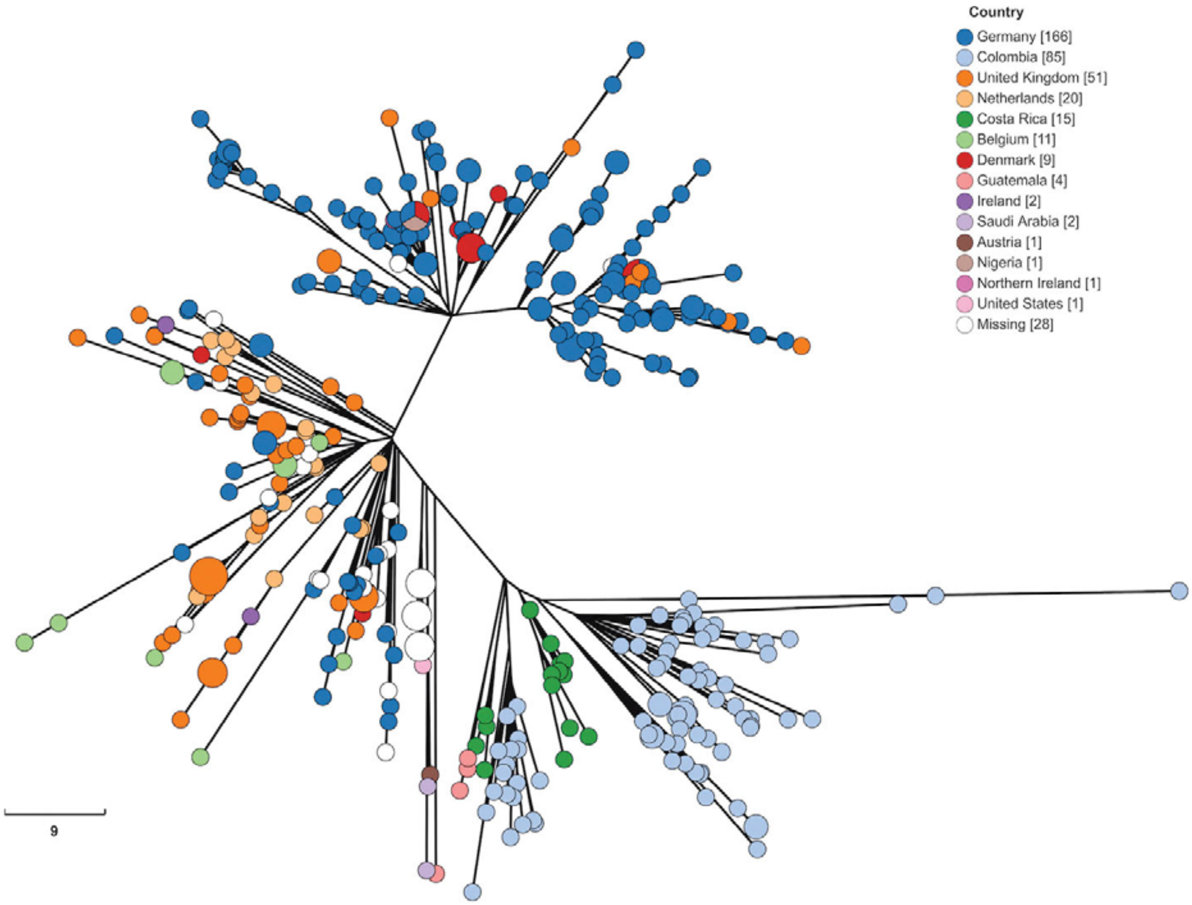

Appendix 2 Figure 3. Minimal spanning tree using the core genome multilocus sequence typing of 397 *Salmonella enterica* serovar Paratyphi B variant Java sequence type 28 genomes available in EnteroBase (accessed January 13, 2020). The tree was made using the GrapeTree tool in EnteroBase (*2*). The country of origin is coded in colors. Scale bar represents the number of loci. The tree shows the separate clustering of historical (Austria and Saudi Arabia), European (Belgium, Denmark, Germany, Ireland, Northern Ireland, the Netherlands, and the United Kingdom), and Latin American (Colombia, Costa Rica and Guatemala) strains.

References

1. Drummond AJ, Rambaut A. BEAST: Bayesian evolutionary analysis by sampling trees. BMC Evol Biol. 2007;7:214. PubMed https://doi.org/10.1186/1471-2148-7-214

2. Alikhan NF, Zhou Z, Sergeant MJ, Achtman M. A genomic overview of the population structure of *Salmonella*. PLoS Genet. 2018;14:e1007261. PubMed https://doi.org/10.1371/journal.pgen.1007261
